# Incidence and risk factors associated with postoperative delirium following primary elective total hip arthroplasty: a retrospective nationwide inpatient sample database study

**DOI:** 10.1186/s12888-020-02742-6

**Published:** 2020-07-01

**Authors:** Qinfeng Yang, Jian Wang, Xusheng Huang, Yichuan Xu, Yang Zhang

**Affiliations:** Department of Orthopaedic Surgery, Division of Orthopaedics, Nanfang Hospital, Southern Medical University, Guangzhou, 1838 Guangzhou Avenue, Guangzhou, 510515 Guangdong China

**Keywords:** Postoperative delirium, Total hip arthroplasty, Nationwide inpatient sample

## Abstract

**Background:**

Postoperative delirium is a common complication following major surgeries, leading to a variety of adverse effects. However, there is a paucity of literatures studying the incidence and risk factors associated with delirium after primary elective total hip arthroplasty (THA) using a large-scale national database.

**Methods:**

A retrospective database analysis was performed based on Nationwide Inpatient Sample (NIS) from 2009 to 2014. Patients who underwent primary elective THA were included. Patient demographics, preoperative comorbidities, length of hospital stay (LOS), total charges, in-hospital mortality, and major and minor perioperative complications were evaluated.

**Results:**

A total of 388,424 primary elective THAs were obtained from the NIS database, and the general incidence of delirium after THA was 0.90%. Patients with delirium after THA presented more preoperative comorbidities, longer LOS, extra hospital charges, and higher in-hospital mortality rate (*P* < 0.001). Delirium following THA was associated with major complications during hospitalization including acute renal failure and pneumonia. Preoperative risk factors associated with postoperative delirium included advanced age, alcohol or drug abuse, depression, neurological disorders, psychoses, fluid and electrolyte disorders, diabetes, weight loss, deficiency anemia, coagulopathy, hypertension, congestive heart failure, valvular disease, pulmonary circulation disorders, peripheral vascular disorders, and renal failure. Both female and obesity were detected to be protective factors.

**Conclusions:**

The results of our study identified a relatively low incidence of delirium after primary elective THA, which is as reported in the NIS and not necessarily the surgical population as a whole. Postoperative delirium of THA was associated with increased preoperative comorbidities, LOS, total charges, in-hospital mortality, and major perioperative complications including acute renal failure and pneumonia. It is of benefit to study risk factors associated with postoperative delirium to moderate its consequences.

## Background

Total hip arthroplasty (THA) remains one of the most successful procedures in alleviating pain and restoring function of the hip [1]. Currently, about 400,000 THAs are performed each year in the United States, which is expected to increase up to 572,000 by 2030 [2]. However, quite a few postoperative patients suffer from postoperative complications.

Postoperative delirium is one of the common complications after THA, resulting in heavy burdens on individuals and society [1]. Delirium is a clinical syndrome characterized by the disturbance of consciousness, cognitive function, or perception. It is regarded to be caused by maladaptation of the brain to the surgical stress [3, 4]. Postoperative delirium is a common complication in geriatric patients after major surgery [5, 6]. The reported incidence of delirium after THA ranges from 5 to 17%, which varies for many reasons, such as patient population, definition and diagnosis of delirium [1, 7–9]. It has become a heavy burden on healthcare resources because it delays discharge and increases medical costs [1, 7, 9]. About 2.4 million hospitalized elderly patients suffer from delirium and the annual cost ranges from $143 billion to $152 billion [10]. Besides, postoperative delirium has an adverse impact on patients, family members, and health care practitioners, as it had been shown to be associated with higher mortality, progressive functional impairment, long-term cognitive disorder and other complications [11–18].

In order to optimize postoperative outcomes and prevent complications, it is critical to identify preoperatively whether patients are at high risk of postoperative delirium [1, 19–21]. Several risk factors associated with postoperative delirium had been reported in previous literatures, among which advanced age is the most frequently acknowledged [1, 9, 19–25]. Other risk factors, including a history of dementia or psychiatric illness, cognitive impairment, polypharmacy, postoperative electrolyte disorders, and diabetes, had also been identified [1, 9, 20–25]. However, currently there is a paucity of studies about the incidence and risk factors associated with postoperative delirium after primary elective THA based on large-scale national database analysis [7, 9, 20–25].

The purpose of this study was to investigate the incidence and risk factors associated with delirium after primary elective THA, based on a national database, with the hypothesis that postoperative delirium has a relatively lower incidence and numerous risk factors to highlight patient groups that might require preoperative optimization. The incidence, patient demographics, Charlson Comorbidity Index (CCI), length of stay (LOS), total charges, in-hospital mortality, major and minor perioperative complications and risk factors associated with postoperative delirium after THA were evaluated.

## Methods

### Data source

The Nationwide Inpatient Sample (NIS) database is part of the Healthcare Cost and Utilization Project, Agency for Healthcare Research and Quality, and was the data source for this study. In the United States, the NIS represents the largest all-payer database of hospital admissions. The NIS collects a stratified sample from more than 1000 hospitals, of approximately 20% of the hospitalizations in the United States each year [26]. The information, including patient demographics, LOS, total hospital charges, diagnostic and procedural codes from International Classification of Diseases (ninth revision) Clinical Modification (ICD-9-CM) were extracted from this database.

### Data collection

Data was obtained from the NIS database from 2009 to 2014. Patients were identified according to ICD-9-CM procedural codes of THA (81.51). Given an inherent limitation that the information about who assessed these patients using which tools at which times was unavailable in NIS database. The diagnoses of delirium might be made by surgeons or physicians varied between hospitals. Hence, delirium only could be defined by ICD-9-CM diagnostic codes based on prior studies. Patients with a diagnosis of delirium were defined by ICD-9-CM diagnostic codes and selected, including transient mental disorders, acute and subacute delirium (293, 293.0, 293.1, 293.8, 293.9, 293.81–84, 293.89), drug-induced delirium (292.81), and altered mental status (780.97) [11]. Patients who were less than 18 years of age, had a hip fracture, were non-elective admission, had osteomyelitis, or had pathologic fracture were excluded from this study.

The recruited cases were divided into two groups according to the occurrence of postoperative delirium. Patient demographics, including age, sex, and race, were evaluated. Outcome measures such as LOS, total charges during hospitalization, and in-hospital mortality were analyzed. Major and minor perioperative complications before discharge were searched from the database by ICD-9-CM diagnostic code. Major perioperative complications were defined as acute renal failure, death, myocardial infarction, pneumonia, pulmonary embolism, and stroke. Minor perioperative complications included deep vein thrombosis, hip dislocation, seroma/hematoma, and wound infection [7]. The included covariates were preoperative comorbidities only and did not include postoperative complications, given a numerical score according to the CCI. There were 17 comorbid conditions assigned with specific point values, where higher score means more comorbidities [27]. As comorbidity definitions in the NIS database vary slightly from the CCI, several modifications were made for analysis: A history of coronary heart disease or leukemia was omitted from the CCI score; liver disease got a weighted value of 2 points instead of 1 point for mild chronic liver disease and 3 points for moderate to severe liver disease [11]. Other comorbid conditions and their point values were: age (age ≤ 50 yrs. = 1, age 51–60 yrs. = 2, age 61–70 yrs. = 3, age ≥ 71 yrs. = 4), congestive heart failure (1), peripheral vascular disorders (1), neurological disorders (1), psychoses (2), chronic pulmonary disease (1), rheumatoid arthritis/collagen vascular diseases (1), peptic ulcer disease (1), diabetes without complications (1), diabetes with complications (2), paralysis (2), renal disease (2), lymphoma (2), liver disease (2), solid tumor without metastasis (2), metastatic cancer (6), and AIDS/HIV (6).

### Data analysis

The statistical software, R version 3.5.3 was used to perform statistical analysis. Significant differences between two groups were determined by Wilcoxon rank test for continuous data and chi-square test for categorical data. Univariate and multivariate logistic regression models were constructed to assess the association of delirium with major and minor perioperative complications. To identify independent risk factors for postoperative delirium, binary logistic regression with the stepwise method was performed. All variables, including demographics, hospital characteristics, and preoperative comorbidities which were provided by the NIS were entered into the regression analysis (Table 1). Statistical significance was defined by an alpha level of *P* ≤ 0.001 because of the large-scale sample volume, which has been utilized by other NIS-researches [11, 28].

**Table 1:** Variables Entered into the Binary Logistic Regression Analysis

Variables Categories	Specific Variables
Patient demographics	Age (≤60 yr and ≥ 61 yr), sex (male and female), race (White, Black, Hispanic, Asian or Pacific Islander, Native American and Other)
Hospital characteristics	bed size of hospital (small, medium, large), teaching status of hospital (nonteaching, teaching), location of hospital (rural, urban)
Comorbidities	AIDS, alcohol abuse, deficiency anemia, rheumatoid arthritis/collagen vascular diseases, chronic blood loss anemia, congestive heart failure, chronic pulmonary disease, coagulopathy, depression, diabetes (uncomplicated), diabetes (with chronic complications), drug abuse, hypertension, hypothyroidism, liver disease, lymphoma, fluid and electrolyte disorders, metastatic cancer, neurological disorders, obesity, paralysis, peripheral vascular disorders, psychoses, pulmonary circulation disorders, renal failure, solid tumor without metastasis, peptic ulcer disease, valvular disease, weight loss

## Results

### Incidence of postoperative delirium in patients undergoing primary elective THA

**Fig. 1:**
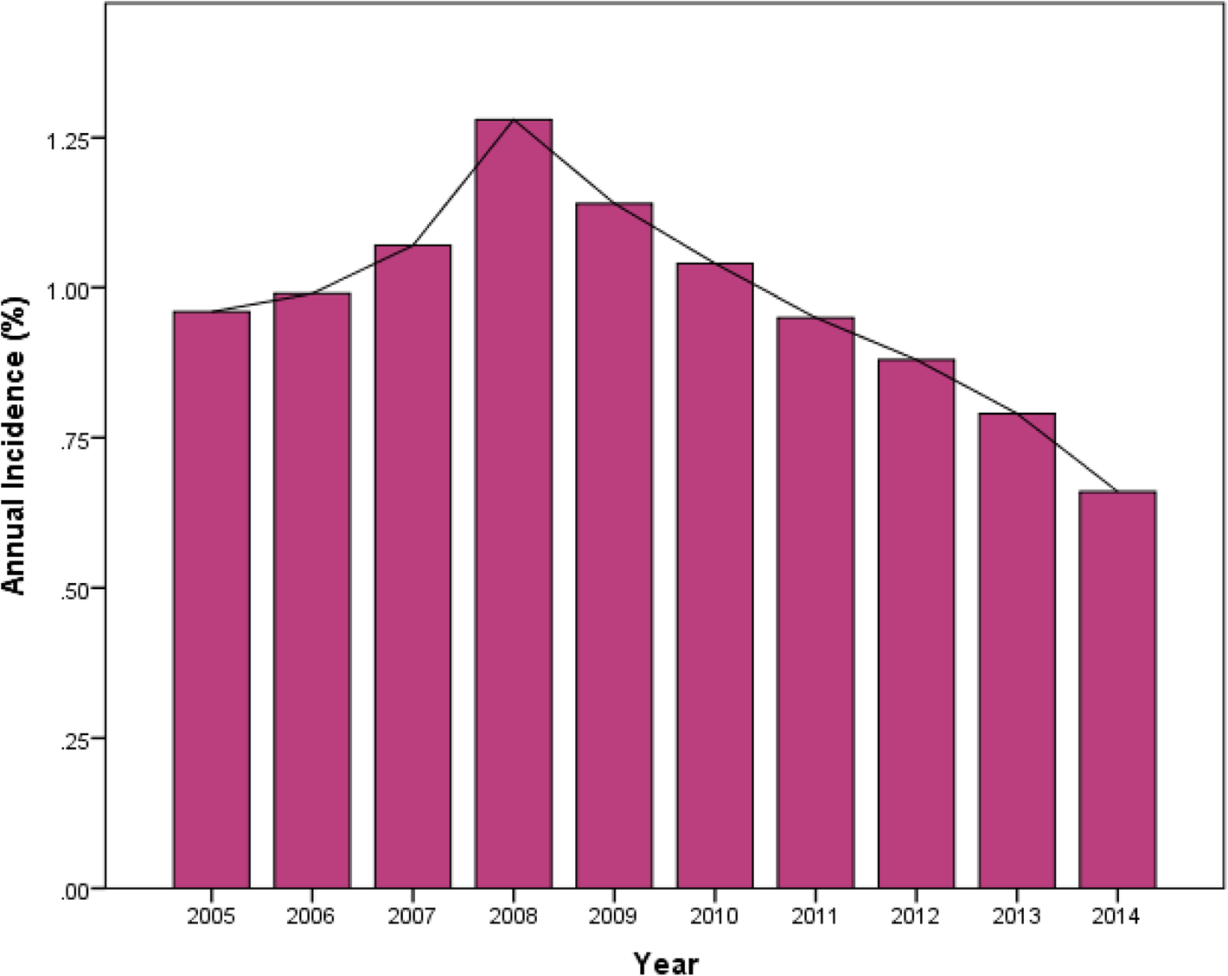
Annual Incidence of Postoperative Delirium in Patients Undergoing Primary Elective THA

It was found that the incidence of postoperative delirium was gradually increasing from 2005 to 2008 (from 0.96 to 1.28%) (Fig. 1), while annually decreasing from 2008 to 2014 (from 1.28 to 0.66%) (Fig. 1). As the incidence clearly changes over time, we chose to focus on the data after break point (2009–2014), which was most current data with a smaller interval to limit heterogeneity.

A total of 388,424 THAs were identified in the NIS database from 2009 to 2014. Overall, there were 3481 cases of postoperative delirium with an incidence of 0.90% (Table 2).

**Table 2:** Patient Characteristics and Outcomes of Delirium after Primary Elective THA (2009–2014)

Parameter	No delirium	Delirium	P
Total (*n* = count)	384,943	3481	
Total incidence (%)	0.90		
Age (yrs.)	65 (57–74)	77 (69–83)	< 0.001
Age group (%)			
≤ 50	11.01	2.15	< 0.001
51–60	23.80	7.07	
61–70	31.19	18.33	
≥ 71	34.00	72.45	
Sex (% female)	55.83	58.16	0.006
Race (%)			
White	86.12	89.52	< 0.001
Black	7.39	5.08	
Hispanic	3.24	2.52	
Asian or Pacific Islander	0.83	0.71	
Native American	0.37	0.32	
Other	2.06	1.84	
CCI^a^	3 (2–4)	4 (4–5)	< 0.001
LOS^b^ (d)	3 (2–3)	4 (3–6)	< 0.001
Total charges ($)	47,936 (35,100.5-66,438)	56,984 (40,339-81,950)	< 0.001
In-hospital mortality (%)	0.13	0.81	< 0.001

*CCI*^*a*^ Charlson Comorbidity Index; LOS^b^: length of stay

### Demographics of patients with postoperative delirium

There was no significant difference of postoperative delirium incidence between two genders (*P* = 0.006). Patients suffered from postoperative delirium (77 yrs.) were significantly older than those without postoperative delirium (65 yrs.) (*P* < 0.001). Consistently, patients older than 71 years accounted for a significant larger proportion in the postoperative delirium group (P < 0.001) (Table 2). Meanwhile, postoperative delirium occurred more frequently in Whites (*P* < 0.001) (Table 2).

### Adverse effects of postoperative delirium after primary elective THA

Patients with postoperative delirium demonstrated significant greater CCI scores (4 vs. 3, *P* < 0.001), which as mentioned previously, represented more comorbidities. Not surprisingly, in-hospital mortality was increased from 0.13 to 0.81% with the presence of postoperative delirium (*P* < 0.001) (Table 2). The mean LOS of patients with delirium was longer than those without delirium (4 d vs. 3 d; P < 0.001) (Table 2). Consequently, postoperative delirium increased medical cost. There was an average increase of $9048 in total hospital charges, with the presence of postoperative delirium ($56,984 vs. $47,936, *P* < 0.001) (Table 2).

Those with delirium were more likely to have major perioperative complications including acute renal failure and pneumonia compared with patients without delirium (P < 0.001) (Table 3). Postoperative delirium were found to be associated with overall major perioperative complications (odds ratio [OR] = 2.59; 95% confidence interval [CI] = 1.92–3.48), and specifically, acute renal failure (OR = 1.95; CI = 1.47–2.59) and pneumonia (OR = 2.48; CI = 1.90–3.25). However, delirium was not associated with any minor perioperative complications (Table 3).

**Table 3:** Complications Associated with Postoperative Delirium Following Primary Elective THA (2009–2014)

Complication	No delirium	Delirium	P	Odds Ratio	95% Confidence Interval
**Major**					
Acute renal failure	8342 (2.17%)	383 (11%)	< 0.001	1.95	1.47–2.59
Death	490 (0.13%)	28 (0.81%)	0.08	1.43	0.96–2.13
Myocardial infarction	74 (0.02%)	6 (0.17%)	0.07	2.20	0.93–5.19
Pneumonia	1904 (0.49%)	127 (3.65%)	< 0.001	2.48	1.90–3.25
Pulmonary embolism	345 (0.09%)	8 (0.23%)	0.42	0.74	0.35–1.55
Stroke	2734 (0.71%)	102 (2.93%)	0.003	1.62	1.18–2.21
Any major complication^a^	12,958 (3.37%)	567 (16.3%)	< 0.001	2.59	1.92–3.48
**Minor**					
Deep vein thrombosis	646 (0.17%)	21 (0.6%)	0.66	0.83	0.36–1.93
Dislocation	857 (0.22%)	30 (0.86%)	0.56	1.29	0.55–3.00
Seroma/hematoma	1010 (0.26%)	48 (1.38%)	0.41	1.39	0.63–3.03
Wound infection	379 (0.1%)	22 (0.63%)	0.35	1.46	0.66–3.21
Any minor complication^b^	2790 (0.72%)	113 (3.25%)	0.03	2.44	1.08–5.50

Any major complication^a^ or minor complication^b^: patients with more than one complication are counted only once

### Risk factors associated with postoperative delirium after primary elective THA

Logistic regression analysis was applied to investigate preoperative risk factors associated with postoperative delirium (Table 4), and the following indicators were identified: advanced age (≥61 years, odds ratio [OR] = 4.27; 95% confidence interval [CI] = 3.76–4.86; *P* < 0.001), alcohol abuse (OR = 1.85; CI = 1.51–2.27), deficiency anemia (OR = 1.32; CI = 1.21–1.45), congestive heart failure (OR = 1.62; CI = 1.42–1.86), coagulopathy (OR = 1.43; CI = 1.22–1.67), depression (OR = 1.39; CI = 1.26–1.53), uncomplicated diabetes (OR = 1.27; CI = 1.15–1.39), diabetes with chronic complications (OR = 1.55; CI = 1.25–1.92), drug abuse (OR = 2.53; CI = 1.90–3.36), hypertension (OR = 1.23; CI = 1.13–1.34), fluid and electrolyte disorders (OR = 2.58; CI = 2.37–2.81), neurological disorders (OR = 9.43; CI =8.69–10.24), peripheral vascular disorders (OR = 1.33; CI = 1.14–1.55), psychoses (OR = 1.82; CI = 1.52–2.17), pulmonary circulation disorders (OR = 1.61; CI = 1.30–2.00), renal failure (OR = 1.40; CI = 1.24–1.58), valvular disease (OR = 1.40; CI = 1.22–1.60), and weight loss (OR = 1.76; CI = 1.41–2.20). Interestingly, there were two protective factors for postoperative delirium including female (OR = 0.88; CI = 0.82–0.95; *P* < 0.001) and obesity (OR = 0.72; CI = 0.64–0.81; *P* < 0.001).

**Table 4:** Risk Factors for Postoperative Delirium (2009–2014)

Variable	Odds Ratio	95% Confidence Interval	P
Age ≥ 61 yr	4.27	3.76–4.86	< 0.001
Female	0.88	0.82–0.95	< 0.001
Alcohol abuse	1.85	1.51–2.27	< 0.001
Deficiency anemia	1.32	1.21–1.45	< 0.001
Chronic blood loss anemia	1.43	1.15–1.79	0.002
Congestive heart failure	1.62	1.42–1.86	< 0.001
Coagulopathy	1.43	1.22–1.67	< 0.001
Depression	1.39	1.26–1.53	< 0.001
Diabetes, uncomplicated	1.27	1.15–1.39	< 0.001
Diabetes with chronic complications	1.55	1.25–1.92	< 0.001
Drug abuse	2.53	1.90–3.36	< 0.001
Hypertension	1.23	1.13–1.34	< 0.001
Lymphoma	1.58	1.04–2.41	0.03
Fluid and electrolyte disorders	2.58	2.37–2.81	< 0.001
Metastatic cancer	1.94	1.27–2.96	0.002
Other neurological disorders	9.43	8.69–10.24	< 0.001
Obesity	0.72	0.64–0.81	< 0.001
Peripheral vascular disorders	1.33	1.14–1.55	< 0.001
Psychoses	1.82	1.52–2.17	< 0.001
Pulmonary circulation disorders	1.61	1.30–2.00	< 0.001
Renal failure	1.40	1.24–1.58	< 0.001
Valvular disease	1.40	1.22–1.60	< 0.001
Weight loss	1.76	1.41–2.20	< 0.001

## Discussion

**Fig. 2:**
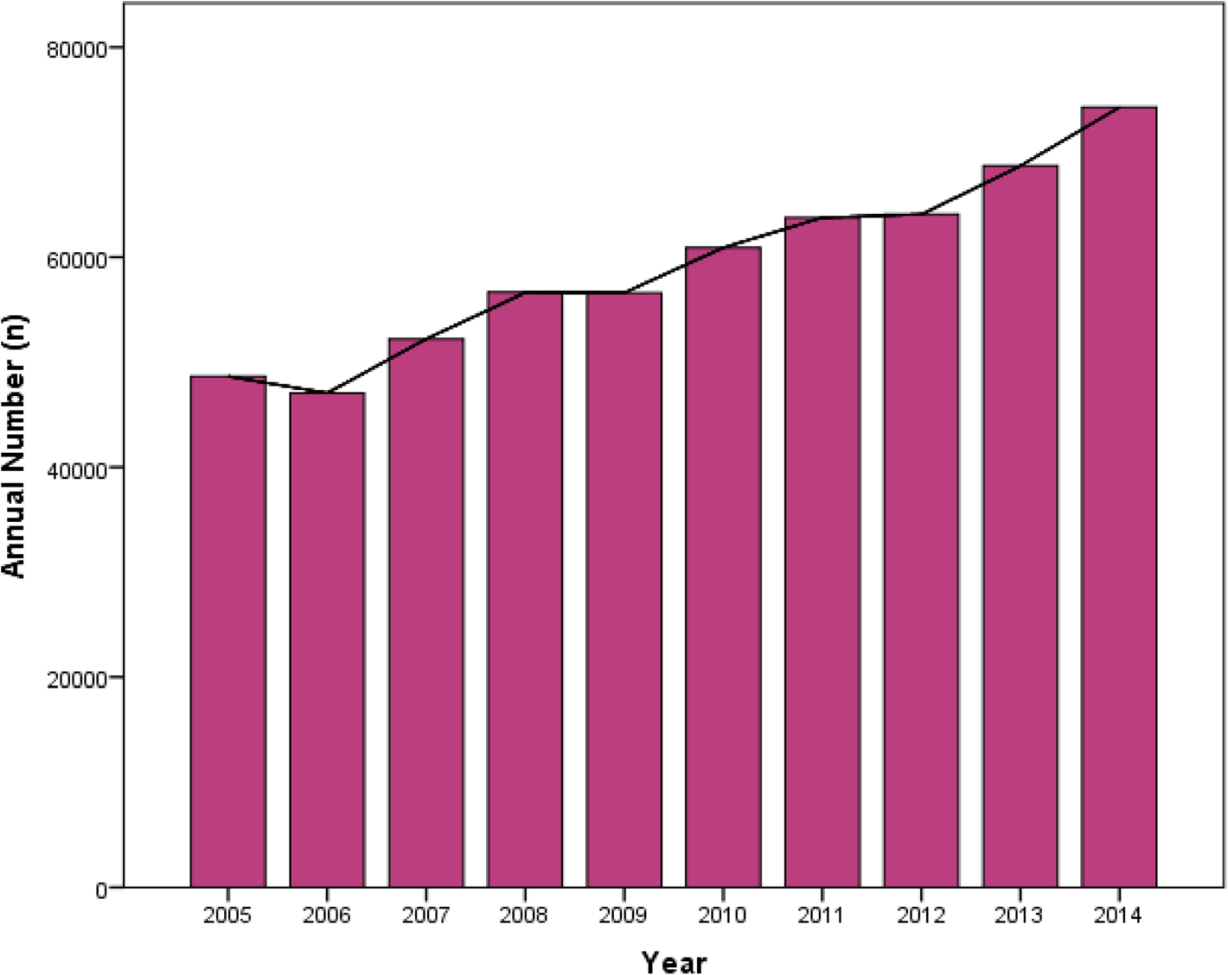
Annual Number of Patients Undergoing Primary Elective THA

This study has provided a large-scale and health-economic analysis of postoperative delirium after primary elective THA. It is worth mentioning that this data represented the NIS and may not be necessarily taken to represent the surgical population as a whole. From the year 2005 to 2008, the incidence of postoperative delirium was increasing annually from 0.96 to 1.28%. Then, the incidence of postoperative delirium decreased gradually to 0.66% in 2014 (Fig. 1). Interestingly, this trend had never been reported in previous studies. Although there was no change of the definition of delirium over this decade, according to ICD-9-CM, the diagnosis of delirium may vary among institutions [11]. One possible explanation accounting for the observed increase in the incidence of delirium following THA prior 2008, to some degree, might be related to growing awareness of this complication by hospital coders [29]. Another possible explanation for this trend may be that the number of THA performed was increased with aging of population (Fig. 2), however, the lack of recognition and medical interventions, or the immature types of anesthesia and protocols of relieving pain likely led to a higher incidence of postoperative delirium [11, 30]. Then postoperative delirium received more and more attention and this trend was reversed after 2008. We identified an overall incidence of 0.90% after THA procedures, which is much lower compared with the previous studies ranging from 5 to 17% [1, 7–9]. There are several possible reasons accounting for this obvious difference. First, previous literatures observed small-scale and selected senior patients, resulting in a higher incidence [11]. Second, the definition and diagnosis of delirium varied among institutions according to the criteria utilized may also contributed to the difference. When the ICD classification is compared with the psychiatric-based Diagnostic and Statistical Manual (DSM) classification, the former is felt to be much more restrictive and less inclusive than the latter [11, 31, 32]. Third, the limitation of NIS database, such as only providing in-hospital delirium, high specificity (low false-positive rate), and low sensitivity (high false-negative rate) may lead to underestimating the incidence [29, 31]. Forth, the actual manifestations of delirium, such as disorganized thinking, alteration of consciousness, cognitive defects, and perceptual disturbances could not be included in NIS because they are not part of an ICD-9-CM diagnostic codes. Furthermore, it is also likely that the hypoactive form of delirium was under-diagnosed [32].

In terms of demographics, patients suffered from postoperative delirium were significantly older than those without. This was highly consistent with previous studies which had identified advanced age as a common independent predictor of postoperative delirium [1, 9, 20–25]. Interestingly, there was significant difference of race distribution between the two groups, indicating a racial difference in occurrence of postoperative delirium. Our study found that Whites patients occupying a larger proportion in the postoperative delirium group. This is consistent with the previous report that Whites patients undergoing general or orthopedic surgery were more likely to develop postoperative delirium [33]. However, very few studies have focused on racial difference in postoperative delirium.

The CCI score of patients with postoperative delirium was significantly higher. This is reasonable as higher CCI score means relatively worse healthy condition before surgery, and may increase postoperative complications including delirium. Postoperative delirium has been reported to increase hospitalization duration, medical cost, and mortality [1, 7, 9, 11]. Similar findings were observed in our study. With the presence of postoperative delirium, the average LOS was 1 day longer and the total hospital charge was $9048 more per admission, which may be due to that these patients need additional nursing instructions and rehabilitation [12, 34]. Further, postoperative delirium may be associated with other postoperative complications [35], including acute renal failure and pneumonia, which was consistent with the observation by Aziz et al. [7].

A systematic review on postoperative delirium after total joint arthroplasty suggested that pre-screening and risk stratification is essential to improve outcomes [1]. Thus, in order to prevent postoperative delirium, it is critical to understand the risk factors before surgery. Logistic regression was applied and the results were consistent with previous publications [1, 9, 20–25]. Interestingly, female and obesity were found to be protective factors. The reasons for these remains unclear but likely multifactorial. There are several prior findings probably relevant and worth discussing [36–38]. Melatonin, a hormone secreted by the pineal gland, has been found to be relevant to female hormone and obesity [36, 37]. Prior study reported that alterations in the metabolism or a disturbed circadian pattern of melatonin may play a role in the development of delirium [38]. The use of melatonin for the prevention of delirium indicates that the complex relationships among female, obesity, melatonin, and postoperative delirium will need further study [36].

Aziz et al. had performed a study of postoperative delirium using the same database from 2000 to 2009 [7]. They also found that delirium was associated with postoperative complications including seroma/hematoma, and wound infection, which was not observed in our study. The possible explanation may be that they studied both total and partial hip arthroplasty, while only primary elective total hip arthroplasty was included in our study. Meanwhile, data from different period (2009–2014 vs. 2000–2009) may also contribute to this inconsistency.

Several limitations exist in utilizing the NIS database. First, information of each patient is only recorded before discharge, meaning any complication that occurs after discharge will not be included in the NIS database. This limitation might contribute to the lower incidence of postoperative delirium as only early period medical records were analyzed [11]. Second, only risk factors recorded in the NIS database could be analyzed. There are other known risk factors that were not available in the NIS database, such as type of anaesthesia, commonly used perioperative medications (opioids, benzodiazepines, and ketamine), sedation during anesthesia recovery, vision impairment, functional impairment, and so on [11, 22, 25, 30]. Third, information about who assessed patients with delirium using which tools at which times was unavailable in NIS database. The diagnoses of delirium might be made by surgeons or physicians varied among hospitals. Hence, delirium only could be defined by ICD-9-CM diagnostic codes based on prior studies [11]. Furthermore, as with any large database, there may be discrepancy or misclassification in coding and documentation. Thus, administrative data tend to have high specificity (low false-positive rate) but low sensitivity (high false-negative rate) in identifying adverse events, which may also underestimate the incidence of postoperative delirium following THA [11, 29, 31].

## Conclusions

Postoperative delirium is a common complication typically occurring in the elderly after primary elective THA, with an overall incidence of 0.90%. The annual incidence of postoperative delirium was increasing gradually from 2005 to 2008 while decreasing from 2008 to 2014. Numerous preoperative risk factors have been identified in this study including advanced age (≥61 yrs.), alcohol and drug abuse, a history of neurological and psychiatric diseases, fluid and electrolyte disorders, complicated or uncomplicated diabetes, weight loss, deficiency anemia, coagulopathy, hypertension, congestive heart failure, valvular disease, pulmonary circulation disorders, peripheral vascular disorders and renal failure. Both female and obesity, however, are protective factors. The occurrence of delirium after THA was associated with an increased LOS, extra total hospital charges, higher inpatient mortality and major perioperative complications (acute renal failure and pneumonia), but not minor complications.

## Data Availability

This study is based on data provided by Nationwide Inpatient Sample (NIS) database, part of the Healthcare Cost and Utilization Project, Agency for Healthcare Research and Quality. The NIS database is a large publicly available all-payer inpatient care database in the United States. Therefore, individual or grouped data cannot be shared by the authors.

## Abbreviations

THA: Total hip arthroplasty
NIS: Nationwide Inpatient Sample
LOS: Length of stay
CCI: Charlson Comorbidity Index
OR: Odds ratio
CI: Confidence interval
